# Advancing Equity and Accessibility in Eyecare

**DOI:** 10.3389/phrs.2025.1606894

**Published:** 2025-03-11

**Authors:** Anthony Obiyom Kamalu, Obinna Princewill Anyatonwu

**Affiliations:** ^1^ Public Health Department, Louisiana State University Shreveport, Shreveport, LA, United States; ^2^ Orchid Foundation for Neurodevelopmental Disabilities, Lagos, Nigeria

**Keywords:** equity, accessibility, eyecare, visual impairment, addressing disparity in eyecare access

Serving as windows to the world, our eyes enable us to perceive and interact with our environment, facilitating our ability to appreciate the beauty of a sunset, read a book, or navigate our daily lives. This establishes vision as one of our most cherished senses; a sense whose importance arguably impacts us the most as it extends to various dimensions of life, wellbeing, sustainability, and the economy [1]. With the importance of vision this well known, it is then sad to know that a considerable number of individuals, families, and communities still endure the negative outcomes resulting from limited access to cost-effective, top-notch eye care, which often results in vision impairments and blindness [1]. This is an unfortunate reality that disproportionately impacts disadvantaged populations, making it crucial to address these inequalities for humanitarian and public health reasons, as well as for economic prosperity.

Around the world today, the absence of adequate eye and vision care for millions of children, low-income workers, and the elderly substantially hinder their ability to learn, perform well at their jobs, access employment prospects, and maintain a safe home environment [2]. As at the time of writing, approximately 2.2 billion individuals are experiencing various levels of visual impairment, most of which could have been avoided [3]. Of this number, 1.8 billion people are grappling with near vision impairments due to presbyopia (both addressed and unaddressed), 252.5 million people are living with moderate to severe vision impairments (due to cataract, myopia, hypermetropia, diabetic retinopathy, trachoma, corneal opacities, glaucoma, age related macular degeneration), 185 million individuals have mild vision impairments due to unknown causes [3–5]. At least 90% of the visual impairments affecting a minimum of 1 billion individuals are entirely avoidable or can be effectively managed with treatment [6].

These figures highlight significant disparities in access to eye care across different regions and socioeconomic groups, with low- and middle-income countries facing a higher prevalence of untreated eye conditions. For example, cataracts and refractive errors, which are among the leading causes of vision impairment, are often left untreated in poorer regions due to a lack of accessible and affordable surgical and corrective services [7]. Conversely, high-income countries like South Korea and China [8, 9] report high myopia rates, likely due to lifestyle factors such as increased screen time and reduced outdoor activities. Preventable vision impairment hampers the advancement of individuals and societies, negatively impacting, education, employment opportunities, productivity and quality of life [10, 11]. This is particularly so for children’s educational prospects and adults' job opportunities, while also affecting the elderly with age-related eye conditions, thus making equitable eyecare access vital.

Eye care service utilization also varies significantly by socioeconomic status, age, gender, and geographic location. A study in Nigeria found that 68% of the participants have never had an eye examination. This low utilization was attributed to significant barriers such as the cost of treatment, perceived need for eye care, waiting times, and distance to clinics [12]. The lack of awareness regarding the importance of regular eye examinations also contributes to this issue [13]. Addressing these multifaceted challenges requires a comprehensive, multi-pronged approach to advance equity and accessibility in eyecare. Firstly, eyecare professionals in public health must prioritize sensitization, awareness creation and public education on eye health. The importance of regular eye examinations can dispel misconceptions and encourage proactive eyecare. This can be done as part of a community-based initiative such as community eyecare programs, mobile clinic events, or in partnership with local healthcare providers. Awareness initiatives go a long way in educating people who lack basic knowledge of eye health and inform them of opportunities available to them.

Affordable vision correction initiatives, such as outreach programs and community eye screenings, offer an additional avenue to enhance accessibility to vision care, particularly for those facing challenges in accessing traditional eyecare settings. In the same breath, equitable access to eyecare may be achieved by leveraging on recent health technology. Novel initiatives such as teleophthalmology, teleoptometry, though not a substitute for traditional care, can facilitate remote eyecare consultations, and may provide access to expert opinions even in remote regions [14]. It allows patients to consult with eyecare providers remotely, particularly in areas with a shortage of eye specialists. Moreover, the growing availability of telehealth services and advanced screening tools has significantly improved early detection of conditions like glaucoma, cataracts, and other eye-related diseases. These developments are particularly beneficial for underserved and disproportionately affected populations, enabling timely diagnosis and intervention, ultimately enhancing patient outcomes and reducing the burden of preventable vision loss [14].

In addition to teleophthalmology and teleoptometry, the presence of AI-based medical devices and the availability of affordable, compact diagnostic equipment such as portable retinal cameras and smartphone applications has emerged as a transformative factor in vision testing. These have eliminated the necessity for extensive infrastructure in delivering eyecare [15]. AI, through its utilization of machine-learning in the field of ophthalmology, can help improve access to eyecare. This is because multiple AI-based retinal grading systems are now available which offer significantly greater speed compared to human graders, consequently diminishing the expenses, workload, and time delays associated with human grading [16]. The presence of AI-based tools such as IDx-DR (an autonomous AI system approved by the U.S. Food and Drug Administration (FDA), for performing on-site screening of diabetic retinopathy, particularly in limited settings), is a game-changer for eyecare providers involved in outreach programs. It enables them to extend their services to a larger number of individuals more efficiently. Moreover, AI’s track record shows improved precision, superior decision-making capabilities, and overall effectiveness. This translates to heightened accuracy and quality in patient care, ultimately benefiting a broader spectrum of people in a timelier manner.

In conclusion, advancing equitable eyecare is not only a moral imperative but also a scientific and economic opportunity. It yields numerous benefits, including improved education, increased workforce productivity, lower healthcare costs, enhanced social inclusion, and an overall better quality of life.
